# Successful Palliative Radiotherapy for Malignant Cardiac Obstruction Caused by Gastric Cancer

**DOI:** 10.7759/cureus.27466

**Published:** 2022-07-29

**Authors:** Teruaki Inoue

**Affiliations:** 1 Department of Internal Medicine, Fujinomiya City General Hospital, Fujinomiya, JPN

**Keywords:** poorly differentiated adenocarcinoma, palliative therapy, gastrointestinal obstruction, gastric cancer, radiotherapy

## Abstract

Gastric cancer is a common malignancy and some patients are diagnosed with an unresectable stage of advanced gastric cancer. Although palliative radiotherapy (RT) is effective for several symptoms in gastric cancer, the duration of efficacy is limited. We report a case where palliative RT significantly improved malignant cardiac obstruction caused by gastric cancer over a long period of time.

A 78-year-old woman was referred to our hospital for anorexia and severe anemia. Esophagogastroduodenoscopy showed the giant gastric tumor in cardia and it was thought to be the cause of anemia. Biopsy revealed poorly differentiated adenocarcinoma; she was diagnosed with gastric cancer. Her clinical cancer staging was IV and she wished to receive palliative care. The malignant cardiac obstruction became severe, and to improve the obstruction, palliative RT was performed. The gastric tumor with a diameter of 4 cm was significantly reduced and the obstruction disappeared. For three years after RT, she had no obstruction symptom.

The sensitivity of cells to radiation is proportional to the degree of differentiation. Palliative RT is effective for several symptoms in gastric cancer, and in our case, the duration of RT efficacy was long. Hence, RT may be useful when the histological type is poorly differentiated in gastric cancer.

## Introduction

Gastric cancer is a common malignancy and one of the major causes of cancer deaths in the world. The standard treatment for resectable gastric cancer is surgery. But some patients who are diagnosed with an unresectable stage of advanced gastric cancer often suffer from bleeding, gastric outlet obstruction and cancer pain. Although several studies have reported the efficacy of palliative radiotherapy (RT) for gastric cancer, the duration of treatment is limited [1-3]. We report a case in which RT significantly improved malignant cardiac obstruction caused by gastric cancer over a long period of time.

## Case presentation

**Figure 1:**
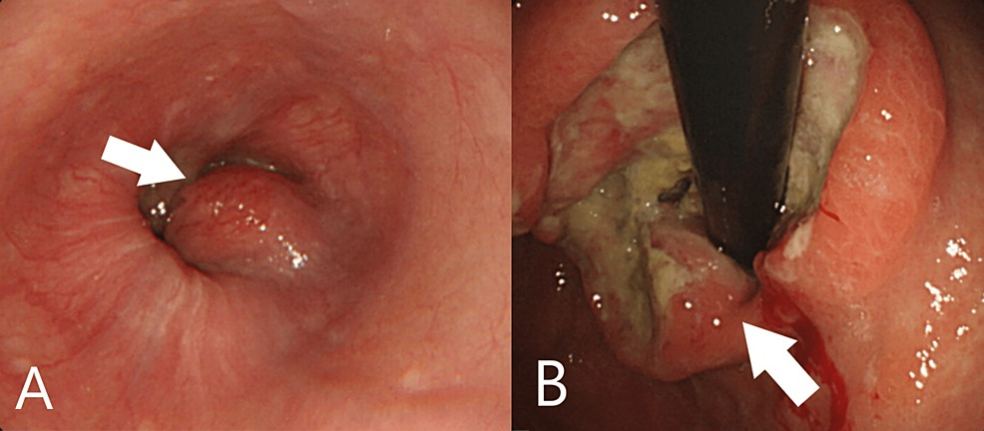
Esophagogastroduodenoscopy images Borrmann type III gastric cancer was confirmed in the cardia (arrow).

**Figure 2:**
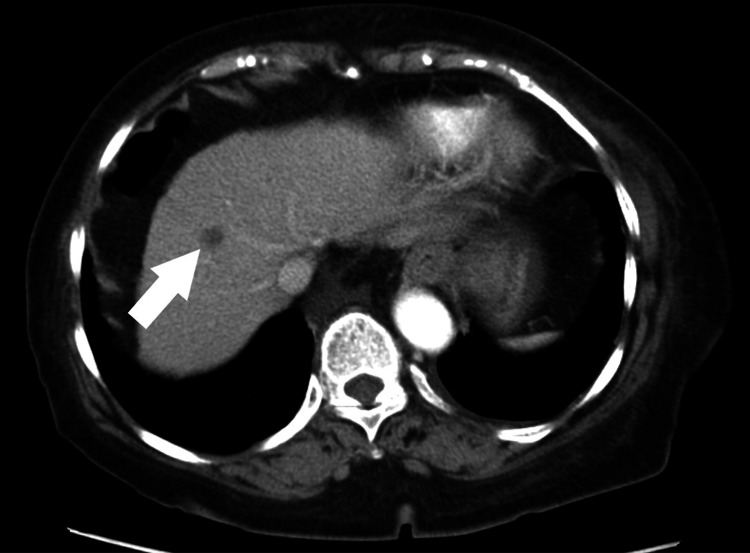
Axial view of the abdominal contrast-enhanced CT scan (arterial phase) One small hypovascular tumor suspected of liver metastasis was confirmed in the liver (arrow).

A 78-year-old Japanese woman was referred to our hospital for anorexia and severe anemia. She had a past medical history of liver cirrhosis and took no significant medication. Upon examination, her vital signs were normal. A blood test showed severe anemia with hemoglobin at 4.4 g/dL. Esophagogastroduodenoscopy (EGD) showed a giant gastric tumor in the cardia and it was thought to be the cause of anemia (Figures 1A, 1B). A biopsy revealed poorly differentiated adenocarcinoma and she was diagnosed with gastric cancer. One small liver metastasis was suspected on computed tomography (CT) and hence her clinical cancer staging became IV (Figure 2). She wished to receive palliative care. Her anemia was improved with blood transfusion, and she was followed up at our hospital.

**Figure 3:**
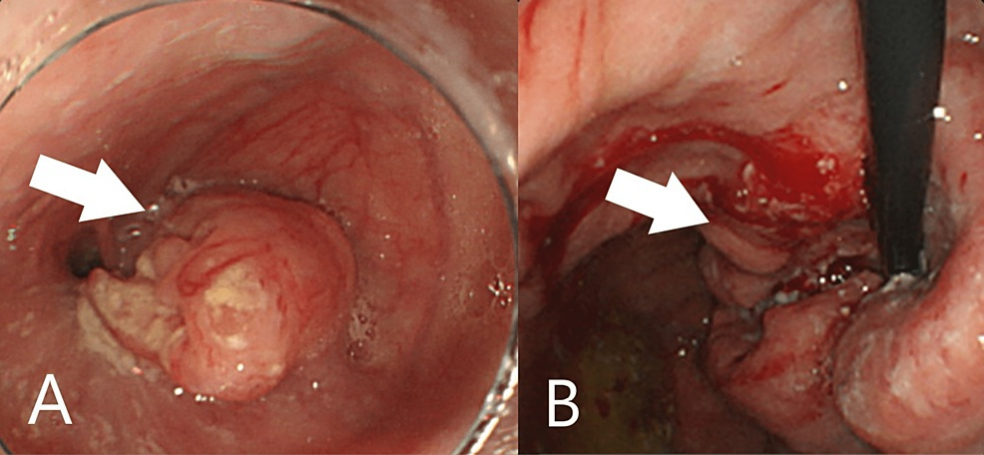
Esophagogastroduodenoscopy images A larger gastric tumor was confirmed in the cardia (arrow).

Three months later, her anemia relapsed and EGD was conducted. EGD revealed that the tumor had grown larger and the GIF-Q260 J endoscope (Olympus, Tokyo, Japan) could not pass through the cardia (Figures 3A, 3B).

**Figure 4:**
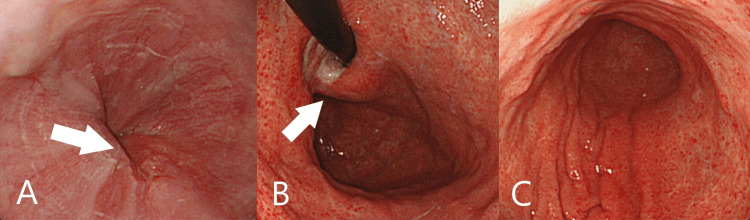
Esophagogastroduodenoscopy done two months after the end of intensity-modulated radiotherapy The gastric tumor in the cardia was significantly reduced (A and B, arrow). Diffuse erythema of the mucosa was confirmed (B and C).

**Figure 5:**
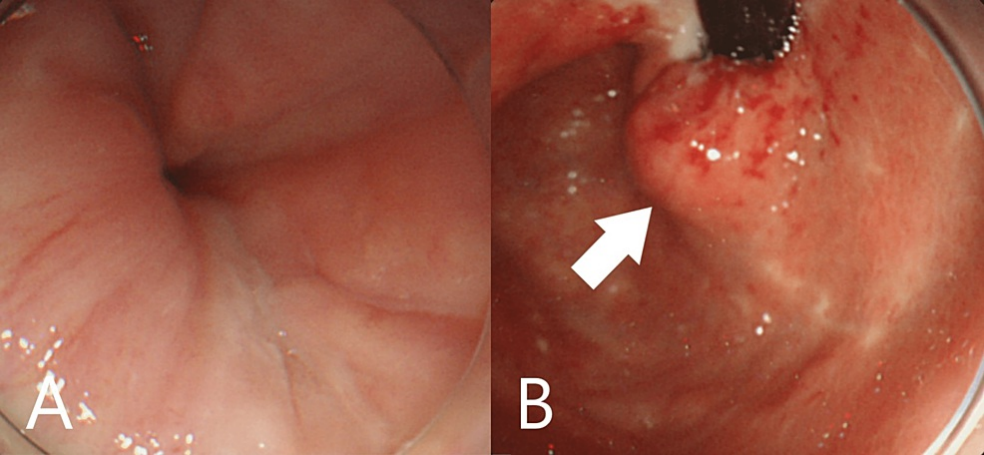
Esophagogastroduodenoscopy done two years after the end of intensity-modulated radiotherapy The gastric tumor had increased slightly, but there was no obstruction in the cardia (A and B, arrow).

Although she had no vomiting and the GIF-XP260N endoscope (Olympus, Tokyo, Japan) could pass through the cardia, the obstruction was severe and she was more likely to be incapable of oral intake. Treatment with a self-expandable metal stent (SEMS) was likely to improve the obstruction, but not thought to improve anemia. Therefore, to improve anemia and the obstruction, palliative RT was performed. She received intensity-modulated radiotherapy (IMRT) of 36Gy in 12 fractions. Blood transfusion improved her anemia, and during IMRT, she had no anemia and was capable of oral intake. Two months after the end of IMRT, she underwent EGD. In EGD, the gastric tumor in the cardia was significantly reduced and the obstruction disappeared (Figures 4A, 4B). In addition, diffuse erythema of the mucosa was confirmed and it was thought to be the radiation-induced gastritis (Figures 4B, 4C). Ten months after the end of IMRT, to treat the single liver metastasis, she received conventional fractionated radiotherapy of 50.4Gy in 28 fractions. However, the liver metastasis did not disappear. Two years after the end of IMRT, the tumor had increased slightly but with no obstruction in the cardia (Figures 5A, 5B). As a result, after the end of IMRT, she often had anemia due to the radiation-induced gastritis, but was capable of adequate oral intake and had no vomiting (Figures 4B, 4C). Three years after the end of IMRT, she died of liver failure due to liver metastasis, with no obstruction symptom.

## Discussion

There are various symptoms in incurable advanced gastric cancer, such as bleeding, gastric outlet obstruction and cancer pain. Palliative therapy for gastric cancer includes medication, RT and gastrectomy. The radiation forms electrically charged particles and deposits energy in the cells of the passing tissue [4]. This energy damages cancer cells by destroying their genetic material. Recently, the efficacy of palliative RT has been reported. Several studies have reported that RT is effective in gastric bleeding, obstruction and pain [1-3,5]. Yu et al. reported that 54 of 61 patients achieved bleeding control using RT [6]. In addition, according to Kim’s study, for RT, the rate of effectiveness for obstruction was 81% [7]. The optimal dose fractionation regimen is unclear and Sapienza et al. reported that there was no association between the number of fractions and bleeding control [8]. Therefore, the short fractionation regimen may be preferable for patients who require urgent symptom control.

Several studies have indicated that the duration of efficacy of treatment is limited, ranging from 1.5 to 11.4 months [3]. However, the sensitivity of cells to radiation is proportional to the degree of differentiation, which means poorly differentiated cells are more radiosensitive [9]. Attia et al. reported that the signet ring cell component (SRCC) is a predictor of the effect of neoadjuvant chemoradiotherapy in rectal cancer [10]. In their study, the SRCC group had a significantly higher rate of complete clinical response and pathologic complete response, compared to the non-SRCC group. In our case, the histological type was poorly differentiated adenocarcinoma and gastric cancer was significantly reduced by RT. To our knowledge, there are no reports on the association between the histological type and RT efficacy in gastric cancer. However, considering the course of disease in our case, there may be a relationship between the histological type and RT efficacy in gastric cancer, as reported by Attia et al., for rectal cancer. Furthermore, Liu et al. reported that microRNA-4537 increases the radiosensitivity of gastric cancer cells [11]. Although genetic information could not be analyzed in this case, there is a possibility that our patient had a gene that increased radiosensitivity.

RT for gastric cancer has several side effects, such as leukopenia, nausea, vomiting and diarrhea [12]. In addition, a serious complication is radiation-induced gastritis [13]. Radiation-induced gastritis usually occurs two to three months after initial radiation and can cause gastrointestinal bleeding. Its endoscopic findings are telangiectasia, mucosal edema and diffuse erythema of the mucosa [13]. In our case, diffuse erythema of the mucosa and telangiectasia were confirmed two months after the end of IMRT (Figures 4B, 4C). Despite the significant reduction in gastric cancer, anemia relapsed, probably due to radiation-induced gastritis. Thus, when RT is conducted, we need to be cautious about it. Treatment for radiation-induced gastritis includes glucocorticoid therapy and argon plasma coagulation [13,14]. However, standard treatment has not yet been established [13].

## Conclusions

In summary, here we have presented a case in which RT significantly improved malignant cardiac obstruction caused by gastric cancer over a long period of time. Palliative RT is effective for several symptoms in gastric cancer, and in our case, the duration of RT efficacy was long. RT may be useful when the histological type is poorly differentiated in gastric cancer.
